# Combinatorial targeting of 2 different steps in adenoviral DNA replication by herpes simplex virus thymidine kinase and artificial microRNA expression for the inhibition of virus multiplication in the presence of ganciclovir

**DOI:** 10.1186/1472-6750-13-54

**Published:** 2013-07-03

**Authors:** Mirza Ibrišimović, Thomas Lion, Reinhard Klein

**Affiliations:** 1 Children’s Cancer Research Institute, St. Anna Kinderkrebsforschung, Zimmermannplatz 10, 1090 Vienna, Austria

**Keywords:** Adenovirus, Vector, RNA interference, MicroRNA, Ganciclovir, HSV-TK

## Abstract

**Background:**

Human adenoviruses are a frequent threat to immunocompromised patients, and disseminated disease is associated with severe morbidity and mortality. Current drugs are not capable of preventing all fatalities, thus indicating the need for alternative treatment strategies. Adenoviruses can be rendered susceptible to antiherpetic prodrugs such as ganciclovir (GCV), upon expression of the herpes simplex virus thymidine kinase (HSV-TK) gene in adenovirus-infected cells. Furthermore, adenoviruses are amenable to post-transcriptional gene silencing via small interfering RNAs (siRNAs) or artificial micro RNAs (amiRNAs).

**Results:**

In this study, we combined these 2 approaches by constructing a combinatorial gene expression cassette that comprises the HSV-TK gene and multiple copies of an amiRNA directed against the mRNA encoding the adenoviral preterminal protein (pTP). HSV-TK gene expression was controlled by the adenoviral E4 promoter, which is activated in the presence of the adenoviral E1 gene products (i.e., when adenovirus is present in the cell). When inserted into a replication-deficient (E1-, E3-deleted) adenoviral vector, this cassette effectively inhibited the replication of wild-type adenovirus *in vitro*. The reduction rate mediated by the combinatorial approach was higher compared to that achieved by either of the 2 approaches alone, and these obvious additive effects became most pronounced when the GCV concentration was low.

**Conclusions:**

The concept presented here has the potential to aid in the inhibition of wild-type adenovirus replication. Furthermore, the combinatorial expression cassette may constitute a safeguard to potentially control unintended replication of adenoviral vectors and to prevent immune responses provoked by them.

## Background

Human adenoviruses are double-stranded (ds) DNA viruses that represent a major risk for immunocompromised patients [1-3], and severe manifestations of adenoviral infections can be life-threatening. Mortality rates as high as 80% have been reported in cases of disseminated disease [4-8]. The incidence of disseminated disease is highest among hematopoietic stem cell transplant recipients, and adenoviruses belonging to species B and C are the main cause of severe adenovirus infections [3,9-11].

Cidofovir (CDV) is the most commonly used agent for the treatment of adenovirus infections. Although the drug demonstrates clinical efficacy, its activity is not sufficient to prevent fatal outcomes in all instances [12-16], and derivatives of CDV are still being evaluated. Thus, alternative strategies to treat severe adenovirus infections have been developed. Donor lymphocyte infusion therapy, and particularly the adoptive transfer of adenovirus-specific T cells represents a promising approach for the treatment of immunocompromised patients [17,18], but its efficacy is still under investigation.

We and others recently investigated the potential of RNA interference (RNAi)-mediated silencing of adenoviral gene expression in the control of the multiplication of adenoviruses *in vitro*[19-22]. RNAi-based approaches to silence viral and non-viral genes employ either the transduction of cells with short interfering RNAs (siRNAs) or the intracellular generation of short hairpin RNAs (shRNAs) and precursors of artificial miRNAs (amiRNAs), respectively, from DNA sequences introduced into those cells [23-28]. In contrast to exogenously added siRNAs, shRNAs and precursor amiRNAs must undergo intracellular processing through the RNAi pathway prior to recognizing their respective target mRNAs and eventually mediating their destruction or causing translational repression. By employing siRNAs directed against a set of adenoviral transcripts required for very different viral processes, genes essential for adenoviral DNA synthesis (i.e., those encoding the preterminal protein (pT) and the viral DNA polymerase) emerged as promising targets for the inhibition of virus multiplication [22]. Furthermore, in a modification of the approach, an amiRNA directed against the pTP mRNA was introduced into wild-type (wt) adenovirus-infected cells via adenoviral vectors [21]. In both approaches, the output of infectious virus progeny from infected cells could be decreased by several orders of magnitude, indicating that RNAi-based methods can, in principle, be employed to control adenovirus replication.

In a very distinct approach, we rendered adenovirus susceptible to treatment with the antiherpetic compound, ganciclovir (GCV), through the targeted expression of the herpes simplex virus thymidine kinase (HSV-TK) gene in wt Ad5-infected cells [29]. GCV is a prodrug that requires phosphorylation by herpes viruses-encoded thymidine kinases to be efficiently converted into its active form [30]. Once activated (i.e., when present in its tri-phosphorylated form), GCV functions as a nucleoside analog, blocking both cellular and viral DNA synthesis by competing with dGTP for incorporation into nascent DNA strands [31,32]. The first phosphorylation step, which is efficiently carried out only by thymidine kinases encoded by herpes viruses (and not by adenoviruses or other viruses), explains the selectivity of GCV for herpes virus-infected cells [33]. Targeted expression of HSV-TK in wt Ad5-infected cells was accomplished by inserting the HSV-TK open reading frame downstream of the Ad5 E4 promoter, whose activity is strongly increased in the presence of the Ad5 E1A gene products (i.e., in cells infected with wt adenovirus) [34,35]. Introducing the HSV-TK expression cassette into wt Ad5-infected cells via a replication-deficient adenoviral vector lacking the E1A region strongly inhibited wt Ad5 DNA replication upon treatment of the cells with low concentrations of GCV, while no obvious effects on viability were observed in cells not infected with wt Ad5.

In the study presented here, we integrated the 2 approaches by generating adenoviral vectors that express both the HSV-TK gene from the adenoviral E4 promoter and, from a distinct expression unit, multiple copies of an amiRNA directed against the wt Ad5 pTP mRNA. We present data indicating that, upon treatment with GCV, the simultaneous expression of both cassettes in wt Ad5-infected cells results in additive anti-adenoviral effects *in vitro*. Moreover, we demonstrate that the additional expression of amiRNAs directed against viral pTP transcripts allows for lower levels of GCV treatment without a loss of antiadenoviral activity. Finally, we discuss how this combinatorial gene expression cassette may be used as a safeguard to potentially control unintended replication of adenoviral vectors and to prevent immune responses provoked by them.

## Methods

### Cell culture, virus amplification, and titer determination

HEK 293 (human embryonic kidney; ATCC CRL-1573), A549 (human epithelial lung carcinoma; ATCC CCL-185), and T-REx-293 cells (Life Technologies Austria, Vienna, Austria) were cultivated in Dulbecco’s Modified Eagle’s Medium (DMEM) with stabilized glutamine (PAA Laboratories, Pasching, Austria) and supplemented with 10% fetal bovine serum (FBS; PAA Laboratories) in a humidified 5% CO_2_ atmosphere at 37°C. Recombinant adenoviral vectors expressing Ad5-directed amiRNAs alone or in combination with the HSV-TK gene were amplified in T-REx-293 cells. Titers of recombinant adenoviruses expressing amiRNAs were determined on T-REx-293 cells by 50% tissue culture infective dose (TCID_50_) assays. Titers of wt Ad5 present in mixed virus suspensions containing both wt and recombinant virus as obtained in co-infection experiments were determined on A549 cells using the same method. All other TCID_50_ assays were performed with HEK 293 cells. Crude virus suspensions for titer determination were obtained by freeze-thawing the samples thrice and removing cell debris by centrifugation at 2800 rpm for 15 min.

### Vector construction

Adenoviral vectors for the combinatorial expression of amiRNAs and HSV-TK were generated by first constructing plasmid vector versions thereof. These “entry” vectors are based on Life Technologie’s Gateway system for recombination-mediated cloning. From these entry vectors, the expression cassettes were eventually transferred into the adenoviral vector backbones via site-specific recombination. All entry vectors for combinatorial amiRNA/HSV-TK expression are based on pEE4_TK [29] and carry the herpes simplex virus 1 thymidine kinase gene downstream of the Ad5 E4 promoter. To generate the combinatorial vectors, the amiRNA expression cassettes were inserted into the *Xmn*I site located downstream of the HSV-TK expression unit. The amiRNA expression cassettes comprise a CMV promoter/enhancer followed by a 2xTetO2 tetracyclin repressor binding site, and end with a BGH poly(A) site. This fragment was obtained by PCR amplification from pcDNA4/TO (Life Technologies Austria, Vienna, Austria) by using primers CMV-TO-f1(5′-TTGCATTTCGAATCTGCTTAGGGTTAGG-3′) and BGHpA-r2 (5′-CCCAGCGAATTCTTTCCGCCTCAGAAG-3′). The *Bcl*I site, located downstream of the promoter/operator region, was used for insertion of the EGFP/amiRNA cassettes, which comprise an open reading frame for EGFP and 1 or 6 copies of either the pTP-mi5 amiRNA or the universal, non-targeting amiRNA inserted into the EGFP 3′UTR. These cassettes were obtained by PCR amplification from vectors pcDNA6.2-GW/EmGFP-miR-neg (Life Technologies; carrying 1 copy of the non-targeting negative control amiRNA), pmiREx6 ([21]; carrying 6 copies of the non-targeting negative control amiRNA), pmiRE-pTP-mi5 ([21]; carrying 1 copy of pTP-mi5), and pmiRE-pTP-mi5x6 ([21]; carrying 6 copies of pTP-mi5) using primers pmiRE-f2 (5′-CAAAAATGATCACTTTAAAACCATGGTGAGC-3′) and pmiRE-r2 (5′- AAGCTGTGATCAGATATCTCGAGTGCGGC-3′). In all amiRNA expression cassettes, the sequences giving rise to pre-amiRNA hairpins are flanked by sequences derived from the murine Mmu-miR-155 pri-miRNA. The final entry vectors were designated pTO-TK-mi- and pTO-TK-mi-×6 (carrying 1 or 6 copies of the non-targeting negative control amiRNA, respectively), and pTO-TK-pTP-mi5 and pTO-TK-pTP-mi5x6 (carrying 1 or 6 copies of pTP-mi5, respectively).

**Figure 1:**
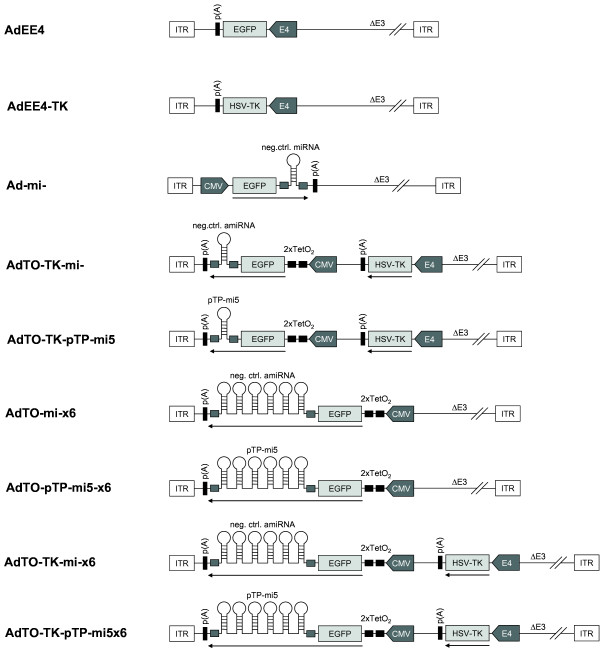
**Schematic representation of adenoviral vectors used in this study.** All vectors were based on the Ad5-derived vector, pAd/PL-DEST™ (Life Technologies), and they lack the E1 and E3 genes. Expression cassettes were inserted into the deleted E1 region in the antisense orientation with respect to the left inverted terminal repeat (ITR). Expression cassettes contained the indicated open reading frames (light grey) for the enhanced green fluorescent protein (EGFP) or the herpes simplex virus 1 thymidine kinase (HSV-TK). Gene expression was driven by the cytomegalovirus promoter (CMV) or the adenoviral E4 promoter (E4). Promoters are indicated as boxed grey arrows. The 2 copies of the tetracycline repressor-binding sequence are indicated as 2xTetO_2_. Mmu-miR-155-derived sequences flanking the hairpins that give rise to mature amiRNAs are indicated as small, dark grey boxes. Polyadenylation sequences are indicated as p(A).

Eventually, the expression cassettes present in the entry vectors were cloned into the deleted E1 region of the adenoviral vector pAd/PL-DEST (Life Technologies Austria, Vienna, Austria), giving rise to the combinatorial adenoviral vectors AdTO-TK-mi-, AdTO-TK-mi-×6, AdTO-pTP-pTP-mi5, and AdTO-pTP-pTP-mi5x6 (Figure 1). This final cloning step was mediated by Life Technologies’ Gateway technology (i.e., by site-specific recombination between sequences flanking the expression cassettes in the entry vectors and the corresponding sequences located in the adenoviral vector). The recombination reaction was performed according to the instructions of the manufacturer (Life Technologies Austria, Vienna, Austria). The construction of the adenoviral vectors AdEE4, AdEE4-TK, Ad-mi-, AdTO-mi-×6, and AdTO-pTP-mi5x6 has been described [21,29].

Restriction enzymes and DNA-modifying enzymes were purchased from Fermentas (St. Leon-Rot, Germany) or New England Biolabs (Frankfurt am Main, Germany). PCR was performed with Pwo DNA polymerase obtained from Roche Diagnostics (Vienna, Austria) or PEQLAB (Erlangen, Germany).

### Nucleic acid extraction

For the extraction of circular plasmid DNA, an EasyPrep Pro Plasmid Miniprep Kit (Biozym, Oldendorf, Germany) or a HiSpeed Plasmid Midi Kit (QIAGEN, Hilden, Germany) was used. PCR products were purified using a QIAquick PCR Purification Kit (QIAGEN, Hilden, Germany), and adenoviral DNA was isolated with a QIAamp DNA Blood Mini Kit (QIAGEN, Hilden, Germany).

### Virus replication experiments

For inhibition of adenovirus replication by siRNA-mediated gene silencing and concomitant HSV-TK expression/GCV treatment, 3e + 04 A549 cells were seeded into the wells of a 96-well plate and transfected with 30 nM siRNA specific for transcripts of the viral DNA polymerase (Pol-si2; sense 5′-CAACGUCUUCCAGCGUCCAACCAUA-3′, antisense 5′-UAUGGUUGGACGCUGGAAGACGUUG-3′), preterminal protein (pTP-si8; sense 5′-GAAAUUGAUUCUGUCGAACUCUCUU-3′, antisense 5′-AAGAGAGUUCGACAGAAUCAAUUUC-3′), IVa2 (IVa2-si2; sense 5′-AAAUACAGUCCAAGAUGCAUCUCAU-3′, antisense 5′-AUGAGAUGCAUCUUCGGACUGUAUUU-3′), hexon (Hex-si2; sense 5′-GAGAACUAAUGGGCCAACAAUCUAU-3′, antisense 5′-AUAGAUUGUUGGCCCAUUAGUUCUC-3′), or protease (Prot si-1; sense 5′-GAGCAGGAACUGAAAGCCAUUGUCA-3′, antisense 5′-UGACAAUGGCUUUCAGUUCCUGCUC-3′). A universal, non-targeting siRNA (Life Technologies) served as a negative control. The functionality of all siRNAs was assessed previously [22]. At 24 h after transfection, the cells were transduced with the adenoviral vectors AdEE4-TK or AdEE4 (Figure 1) at an MOI of 100 TCID_50_/cell. At 24 h after transduction, the cells were infected with Ad5 at an MOI of 0.01 TCID_50_/cell and cultivated in the presence or absence of 1.2 μM GCV for an additional 2 days before extraction of DNA and determination of wt Ad5 genome copy numbers.

Inhibition of adenovirus replication by amiRNAs and/or HSV-TK expression/GCV treatment was assessed by seeding 3e + 04 A549 cells into the wells of 96-well plates, followed by transduction with the adenoviral vectors encoding HSV-TK and one or more amiRNA copies at an MOI of 100 TCID_50_/cell. After 24 h, the cells were infected with wt Ad5 at an MOI of 0.01 TCID_50_/cell and cultivated in the presence of GCV at concentrations ranging between 0 and 1.2 μM for up to 6 days. The cultures were subjected either to DNA isolation for determination of wt Ad5 genome copy numbers or to TCID_50_ analysis.

The inhibitory effect of the HSV-TK/amiRNA expression cassette on the replication of vector AdTO-TK-pTP-mi5x6 was determined by transducing 1.2e + 05 T-REx-293 cells with the vector at an MOI of 0.01 TCID_50_/cell. At the time of transduction, amiRNA expression was induced by adding 1 μg/ml doxycycline to the medium, and GCV was added at concentrations ranging between 0 and 1.2 μM. At 48 h after transduction, the cultures were subjected to DNA isolation and determination of vector copy numbers by qPCR.

### Determination of adenovirus genome copy numbers

Determination of wt Ad5 DNA levels was performed by qPCR using a TaqMan primer/probe set specific for the E1A gene (E1A-fwd 5′-GACGGCCCCCGAAGATC-3′, E1A-rev 5′-TCCTGCACCGCCAACATT-3′, and E1A-p 5′-CGAGGAGGCGGTTTCGCAGA-3′). For the determination of DNA levels of recombinant adenoviral vectors, a TaqMan primer/probe set specific for the hexon gene was used (hexon-fwd 5′- CACTCATATTTCTTACATGCCCACTATT-3′, hexon-rev 5′-GGCCTGTTGGGCATAGATTG-3′, hexon-probe 5′-AGGAAGGTAACTCACGAGAACTAATGGGCCA-3′). Adenovirus genome copy numbers were determined using serial dilutions of an adenoviral reference DNA as a standard.

### FACS analysis

EGFP expression rates were determined by FACS analysis. Cells transduced with EGFP-expressing adenoviruses were harvested by trypsinization, resuspended in normal cell culture medium, and pelleted by centrifugation at 1200 rpm for 5 min. Thereafter, cells were washed once with phosphate buffered saline (PBS) and fixed with 1% formaldehyde in PBS. Samples were analyzed with a FACS Calibur analyzer (Becton Dickinson, Heidelberg, Germany), and percentages of fluorescent cells and mean fluorescence intensities (MFIs) were calculated.

### Statistical analysis

All the data are expressed as mean ± standard deviation (SD). In cases in which the dataset consisted of only 2 groups of samples, Student’s *t*-test was applied to test for statistical significance. For the analysis of larger datasets, one-way ANOVA, corrected with Bonferroni´s post-hoc test, was applied. A *p*-value less than 0.05 was considered statistically significant.

## Results

### Adenovirus-directed siRNAs increase the HSV-TK/GCV-mediated anti-adenoviral effect

We have previously shown that siRNAs or adenoviral vector-encoded amiRNAs targeting viral mRNAs coding for essential viral DNA synthesis components (i.e., the viral DNA polymerase and pTP, respectively) can inhibit wt Ad5 replication *in vitro*[21,22]. We have also demonstrated that the targeted expression of HSV-TK in wt Ad5-infected cells renders adenovirus amenable to inhibition by GCV, through the suppression of viral DNA synthesis [29]. Thus, it is conceivable that a combination of the 2 approaches can lead to additive effects.

**Figure 2:**
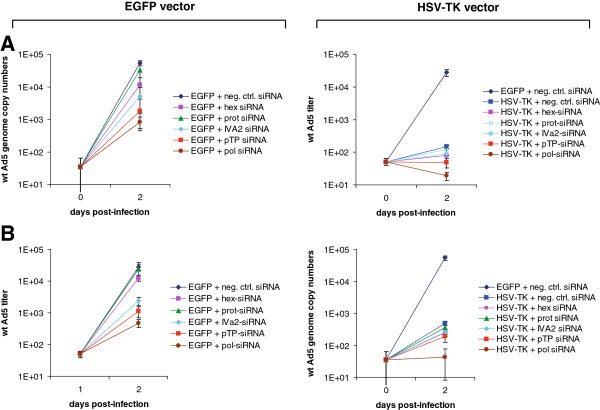
**A combination of HSV-TK expression/GCV treatment and siRNA-mediated silencing of adenoviral gene expression leads to an additive inhibitory effect on adenoviral DNA replication.** A549 cells were transfected with 30 nM siRNA directed against the hexon (hex), protease (prot), IVa2, pTP, and viral DNA polymerase (pol) transcripts. A nontargeting siRNA served as a negative control. At 24 h after transfection, cells were transduced with either the adenoviral HSV-TK expression vector, AdEE4-TK (right panels), or the analogous EGFP expression vector, AdEE4 (left panels), at an MOI of 100 TCID_50_/cell. Another 24 h later, cells were infected with wt Ad5 at an MOI of 0.01 TCID_50_/cell, and GCV was or was not added at a concentration of 1.2 μM. At 48 h after infection with wt Ad5, the cultures were analyzed for wt Ad5 multiplication. **A**. Genome copy numbers were determined by qPCR using a primer/probe set specific for the E1A gene present only on wt Ad5. **B**. The output of infectious virus progeny was determined by TCID_50_ assays. Data represent the means ± SD of 3 independent transfections/infections.

To obtain evidence for such additive effects, we transfected A549 cells with the panel of siRNAs directed against the hexon, viral protease, IVa2, pTP, and viral DNA polymerase mRNAs selected in the previous study. Subsequently, cells were transduced with the adenoviral HSV-TK expression vector, AdEE4-TK, or its respective negative control vector, pADEE4 carrying an EGFP gene instead of the HSV-TK gene, and were treated with 1.2 μM GCV. This concentration is in the range of patient serum levels after treatment with typical doses of GCV [36-38], and has previously been shown by us to inhibit wt Ad5 replication in cells expressing HSV-TK from AdEE4-TK, while leaving cells not infected with wt Ad5 unaffected [29]. Finally, cells were infected with wt Ad5, and 48 h after infection, wt Ad5 genome copy numbers were determined. Transfection of siRNA alone inhibited wt Ad5 replication to an extent comparable to that obtained in our previous study (Figure 2A, left panel). As already demonstrated, siRNAs targeting early transcripts (among them the pTP and DNA polymerase mRNA-targeting siRNAs) were much more efficient than those targeting late transcripts (i.e., those coding for the hexon protein and the viral protease). The highest inhibition rates were obtained with the DNA replication-targeting anti-pTP and anti-DNA polymerase siRNAs, with the latter leading to an inhibition rate of 2 orders of magnitude (Figure 2A, left panel). Alternatively, HSV-TK expression alone decreased wt Ad5 genome copy numbers by 2.3 orders of magnitude (Figure 2A, right panel). However, wt Ad5 genome copy numbers declined even further upon concomitant transfection of cells with the siRNAs (Figure 2A, right panel). Again, the viral DNA replication-affecting siRNAs led to the most prominent additive effects. These effects were not only visible as decreased wt Ad5 genome copy numbers, but also as a reduction in the output of infectious virus progeny (Figure 2B).

### Combined HSV-TK and amiRNA expression increases the anti-adenoviral effect in the presence of GCV

These results prompted us to generate a combinatorial adenoviral vector harboring the HSV-TK expression unit, such as that present on AdEE4-TK, and an amiRNA expression cassette, as found in AdTO-pTP-mi5. In our previous study [21], an amiRNA (pTP-mi5) targeting the Ad5 pTP mRNA was identified as the most potent amiRNA for inhibition of wt Ad5 DNA replication *in vitro*. Thus, in the present study, we merged the adenoviral HSV-TK expression vector, AdEE4-TK, with the adenoviral pTP-mi5 expression vector, AdTO-pTP-mi5, to create the adenoviral vector AdTO-TK-pTP-mi5 (Figure 1). A corresponding negative control vector (AdTO-TK-mi-) carrying an expression cassette for a negative control amiRNA instead of pTP-mi5 was also constructed. We decided to use replication-deficient adenoviral vectors for the combined HSV-TK/amiRNA expression and delivery, because this type of vector may prove advantageous in an envisioned therapeutic application. Because of the shared organ tropism of the adenoviral vector and the wt virus, such a vector may ensure the delivery of the expression cassettes into those cells that are also the preferred targets of the wt virus.

**Figure 3:**
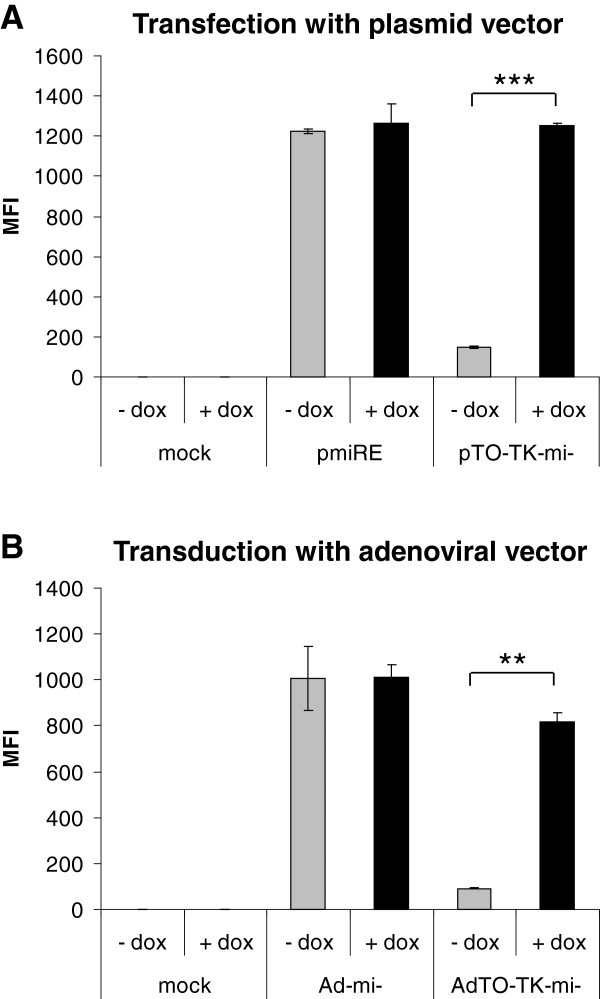
**Expression of the EGFP/amiRNA cassette from the tetracycline repressor-controlled CMV promoter is inhibited in adenovirus packaging cells in the absence of doxycycline. A**. T-REx-293 cells were transfected with the plasmid vector, pTO-TK-mi-, in which the expression of the EGFP/negative control amiRNA expression cassette from the CMV promoter is under control of the tetracycline repressor. The plasmid vector, pcDNA6.2-GW/EmGFP-miR-neg (abbreviated as pmiRE), in which the same cassette is expressed from a constitutive CMV promoter, was used as a control. The cells were incubated with or without doxycycline and, 24 h later, they were analyzed for EGFP expression by FACS analysis. Mean fluorescence intensities (MFIs) of a representative experiment performed in triplicate (mean ± SD; n = 3) are shown; ***p < 0.001. **B**. Setup and data acquisition as in (A), except that cells were transduced with the adenoviral versions of the vectors that allow either constitutive expression of the EGFP/amiRNA cassette (Ad-mi-) or tetracycline repressor-controlled expression (AdTO-TK-mi-). **p < 0.01.

**Figure 4:**
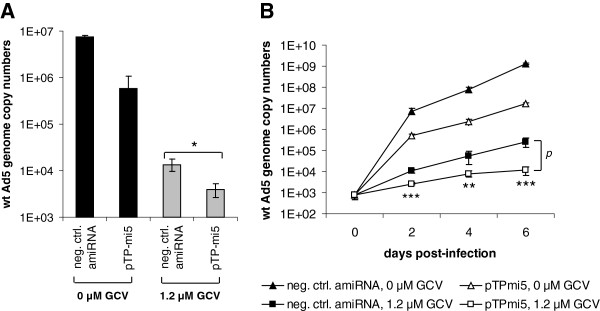
**A combination of HSV-TK expression and pTP-mi5 expression leads to an additive inhibitory effect on adenoviral DNA replication upon treatment with GCV.** A549 cells were transduced with the adenoviral HSV-TK/pTP-mi5 expression vector, AdTO-TK-pTP-mi5, or the respective control vector, AdTO-TK-mi-, expressing a negative control amiRNA at an MOI of 100 TCID_50_/cell. At 24 h post-transduction, cells were infected with wt Ad5 at an MOI of 0.01 TCID_50_/cell, and GCV was or was not added at a concentration of 1.2 μM. **A**. Wt Ad5 genome copy numbers were determined at 48 h post-infection with wt Ad5 by qPCR using a primer/probe set specific for the E1A gene present only on wt Ad5. **B**. The infection kinetics for wt Ad5 was monitored over a time span of 6 days by determining genome copy numbers in the same way as described for (**A**). Data represent the means ± SD of 3 independent transductions/infections. *p < 0.05, **p < 0.01, ***p < 0.001.

Because efficient amplification of adenoviral vectors containing an adenoviral DNA replication-targeting amiRNA cassette in packaging cells requires the shutdown of amiRNA expression in these cells, amiRNA expression in our system is under the control of a tetracycline repressor/operator system. When produced in packaging cells, the tetracycline repressor shuts down amiRNA expression. As demonstrated in Figure 3, the regulatable expression system is functional in both the plasmid and adenoviral vectors constructed in this study: the production of EGFP, which can serve as a measure of amiRNA production because it is encoded by the same primary transcript that gives rise to the amiRNA (see Figure 1), was strongly suppressed in packaging cells upon expression of the tetracycline repressor. To investigate whether the combinatorial vector leads to increased inhibition of wt Ad5 replication, we transduced A549 cells with AdTO-TK-pTP-mi5 or the negative control vector, AdTO-TK-mi-, and infected them with wt Ad5. Concomitantly, cells were or were not treated with 1.2 μM GCV, and wt Ad5 genome copy numbers were determined 48 h post-infection with wt Ad5 (Figure 4A). In the absence of GCV, pTP-mi5 expression inhibited wt Ad5 replication by slightly more than 1 order of magnitude. HSV-TK expression in the presence of 1.2 μM GCV but in the absence of the pTP-targeting amiRNA led to a decrease of wt Ad5 genome copy numbers by 2.7 orders of magnitude. However, a combination of pTP-mi5 and HSV-TK expression and concomitant treatment with GCV led to a further significant drop in wt Ad5 genome copy numbers. Neither the negative control amiRNA nor the expression of HSV-TK in the absence of GCV significantly inhibited wt Ad5 replication (data not shown). The additive effect of concomitant pTP-mi5 and HSV-TK expression became more prominent when we cultivated the wt Ad5-infected cells for longer periods, and measured wt Ad5 genome copy numbers at 6 days post-infection with wt Ad5 (Figure 4B). Here, the additional gain in the inhibition rate was greater than one order of magnitude.

### Multicopy amiRNA/HSV-TK expression inhibits wt adenovirus replication even at very low GCV concentrations

**Figure 5:**
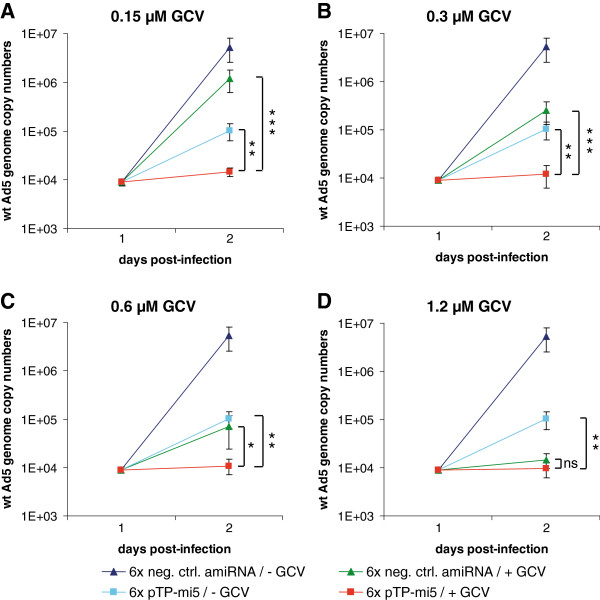
**Improvement of the amiRNA expression cassette by concatemerization of amiRNA hairpins and inhibition of adenoviral DNA replication at decreased GCV concentrations.** A549 cells were transduced with the adenoviral HSV-TK/pTP-mi5 expression vector, AdTO-TK-pTP-mi5×6 (carrying 6 copies of pTP-mi5), or the respective control vector, AdTO-TK-mi-×6 (carrying 6 copies of a negative control amiRNA) at an MOI of 100 TCID_50_/cell. At 24 h post-transduction, cells were infected with wt Ad5 at an MOI of 0.01 TCID_50_/cell, and GCV was or was not added at concentrations of 0.15 μM (**A**), 0.3 μM (**B**), 0.6 μM (**C**), or 1.2 μM (**D**). Wt Ad5 genome copy numbers were determined at 48 h post-infection with wt Ad5 by qPCR using a primer/probe set specific for the E1A gene present only on wt Ad5. Data represent the means ± SD of 3 independent transduction/infection experiments, each performed in triplicate. In each panel, data points for cells that were not treated with GCV are given as a reference. **p < 0.01, ***p < 0.001.

To create the final version of the vector, we improved the amiRNA/HSV-TK expression cassette by incorporating additional copies of pTP-mi5 hairpins into the EGFP/amiRNA transcription unit. This approach had previously resulted in increased knockdown of pTP gene expression [21] and hence, it was anticipated to be beneficial for the function of the vectors generated in this study. The final vectors (i.e., AdTO-TK-pTP-mi5×6 and its analog, AdTO-TK-mi5-×6, carrying the non-targeting amiRNA expression cassette) were used to transduce A549 cells. Concomitantly, the cells were treated with GCV and infected with wt Ad5, as performed previously. Because HSV-TK expression and concomitant treatment with 1.2 μM GCV alone had already been proven to efficiently inhibit wt Ad5 replication, we assumed that additional expression of the 6xpTP amiRNA cassette might allow for efficient inhibition of wt Ad5 replication at even lower GCV concentrations. Thus, we incubated the cells with increasing amounts of GCV (ranging from 0.15 to 1.2 μM) and determined wt Ad5 genome copy numbers at 48 h post-infection (Figure 5). As expected, at the highest GCV concentration of 1.2 μM, the additive effect of 6xpTP-mi5 expression on wt Ad5 inhibition was minor and, in the particular set of experiments shown here, not statistically significant (Figure 5D; compare green and red lines). However, the gain in wt Ad5 inhibition became clearly visible upon gradually lowering the GCV concentration (Figure 5A to C). In fact, even at the lowest concentration of 0.15 μM (Figure 5A), the total inhibition rate (~2.7 orders of magnitude) was comparable to that after treatment with the almost 10-fold higher GCV concentration of 1.2 μM in Figure 5D (compare red lines in Figure 5A to D). In contrast, the inhibition rate gradually decreased upon lowering the GCV concentration in the presence of the non-targeting negative control amiRNA (compare green lines in Figure 5A to D). In conclusion, simultaneous expression of the 6xpTP-mi5 expression cassette allowed for a 10-fold decrease in the GCV concentration, without resulting in a significant loss in the inhibition rate. At any GCV concentration, the combinatorial inhibitory effect was also clearly higher than the effect that was mediated by the 6×pTP-mi5 expression cassette alone (blue line in Figure 5A to D).

**Figure 6:**
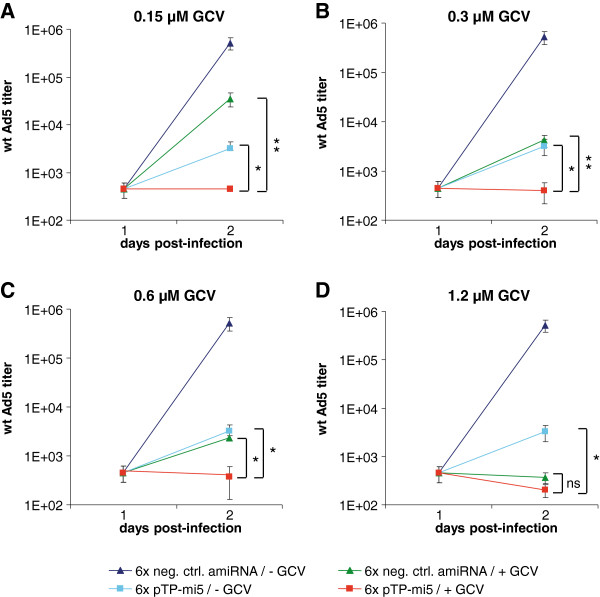
**The concomitant expression of pTP-mi5 and HSV-TK results in an increased inhibitory effect on the generation of infectious virus progeny in the presence of GCV.** A549 cells were transduced with the adenoviral HSV-TK/pTP-mi5 expression vector, AdTO-TK-pTP-mi5x6 (carrying 6 copies of pTP-mi5), or the respective control vector, AdTO-TK-mi-x6 (carrying 6 copies of a negative control amiRNA) at an MOI of 100 TCID_50_/cell. At 24 h post-transduction, cells were infected with wt Ad5 at an MOI of 0.01 TCID_50_/cell, and GCV was or was not added at concentrations of 0.15 μM (**A**), 0.3 μM (**B**), 0.6 μM (**C**), or 1.2 μM (**D**). The output of infectious virus progeny was determined by TCID_50_ assays. Data represent the means ± SD of 3 independent transduction/infection experiments each performed in triplicate. *p < 0.05, **p < 0.01.

The additive effect mediated by the 6×pTP-mi5 expression unit also manifested as a further drop in the output of infectious virus progeny as determined by TCID_50_ analysis (Figure 6). Again, this effect became most pronounced when GCV became limiting (i.e., at GCV concentrations < 1.2 μM) (compare red lines in Figure 6A to C with that in Figure 6D).

### The combinatorial HSV-TK/amiRNA expression cassette inhibits adenoviral vector replication

**Figure 7:**
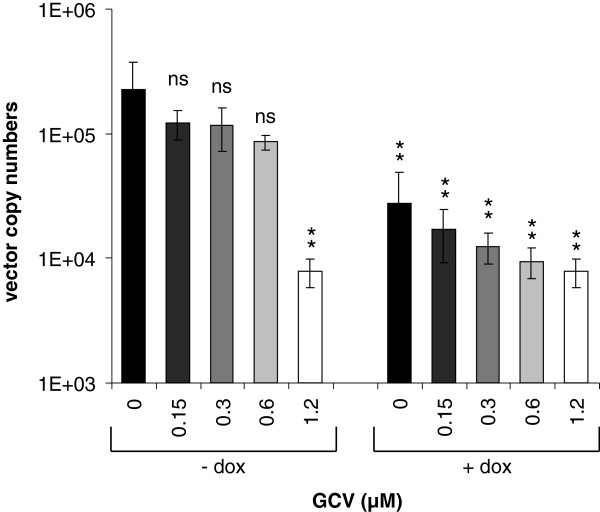
**The adenoviral vector containing both the pTP-mi5 and HSV-TK expression cassettes is heavily compromised in its replication rate in the presence of the E1 gene products.** T-REx 293 cells that express the adenoviral E1 genes were transduced with AdTO-TK-pTP-mi5x6 at an MOI of 0.01 TCID_50_/cell. At the same time, amiRNA expression was or was not induced by the addition of 1 μg/ml doxycycline (dox) to the medium, and GCV was added at concentrations ranging between 0 and 1.2 μM. Vector copy numbers were determined at 48 h post-transduction by qPCR using a primer/probe set specific for the hexon gene present on AdTO-TK-pTP-mi5x6. Data represent the means ± SD of 3 independent transductions. Statistically significant differences between the individual samples and the -dox/0 μM GCV control sample are indicated. **p < 0.01; ns, not significant.

In the presence of GCV and in the absence of the tetracycline repressor, the combinatorial expression cassette comprising the EGFP/amiRNA and HSV-TK transcription units should not only have a negative effect on the replication of a wt adenovirus present in the same cells, but also on the adenoviral vector itself by which it is carried. To investigate this inhibitory effect, we transduced T-REx 293 cells, which carry the adenoviral E1 genes and thus promote the replication of otherwise replication-deficient adenoviral vectors, with the combinatorial vector, AdTO-TK-pTP-mi5x6. We cultivated the cells with or without doxycycline for an additional 48 h to determine the amiRNA-mediated inhibitory effect, and treated them with increasing amounts of GCV (ranging between 0 and 1.2 μM) to investigate the HSV-TK-mediated effect. GCV treatment alone, in the absence of pTP-mi5 expression (i.e., cells not treated with doxycycline), was only effective at the highest GCV concentration of 1.2 μM (Figure 7). Here, the amount of vector DNA was decreased by ~1.5 orders of magnitude. No significant inhibition was seen at lower concentrations of GCV, ranging from 0.15 to 0.6 μM. The expression of the pTP-mi5 amiRNA alone (doxycycline-treated cells, 0 μM GCV) decreased vector copy numbers by about one order of magnitude. The expression of pTP-mi5 in addition to GCV treatment led to a clear increase in the overall inhibitory effect when low concentrations of GCV were used (compare –dox samples with + dox samples). Thus, in agreement with the previous results (Figure 5), the combinatorial amiRNA/HSV-TK expression cassette was of the highest benefit when GCV was limiting. Leaky expression from adenoviral promoters is an issue in the application of adenoviral vectors as gene delivery vehicles, and recombination with wt adenovirus may transform replication-deficient, E1- and E3-deleted adenoviral vectors into replication-competent versions. Thus, taken together, the combinatorial HSV-TK/amiRNA expression cassette presented here may constitute a tool for the decrease of adenoviral background gene expression and aid in the control of unintended vector replication, or, when present in cells that become infected with wt adenovirus, may inhibit wt adenovirus replication and spreading.

## Discussion

We previously demonstrated the inhibition of adenovirus replication both by RNAi-based methods and through the targeted expression of HSV-TK in adenovirus-infected cells with concomitant GCV treatment. Both approaches targeted viral DNA replication, albeit in different ways. While the siRNAs/amiRNAs decreased the number of functional viral proteins that are needed for the initiation (pTP) or progression of viral DNA replication (DNA polymerase), GCV-ppp acted downstream of these steps in a functionally different way (i.e., as a nucleoside analog by preventing the DNA polymerization process). CDV is mechanistically related to GCV-ppp since it blocks the same step during virus replication [39], and we have demonstrated that the expression of a pTP RNA-targeting amiRNA in adenovirus-infected cells and concomitant treatment with CDV leads to additive inhibitory effects [21]. Thus, it was conceivable that a combination of pTP gene silencing via an amiRNA and HSV-TK expression/GCV treatment can result in a similar effect. This assumption was supported by results demonstrating that siRNAs targeting viral transcripts required for DNA replication increased the HSV-TK/GCV-mediated effect (Figure 2). In these experiments, we did not only include the siRNAs with the highest previously proven inhibitory effect on adenoviral DNA replication, but also the ones that had resulted in poor antiviral effects in our previous study (i.e., the hexon and viral protease RNA-targeting siRNAs). We hypothesized that a strong inhibition of viral DNA replication by HSV-TK expression/GCV treatment (which is more effective than that mediated by viral DNA polymerase or pTP mRNA-targeting siRNAs) should decrease viral DNA genome copy numbers and, consequently, hexon and protease gene copy numbers. This, in turn, should result in a decrease in the otherwise vast amounts of hexon and protease transcripts present in adenovirus-infected cells, which may allow the siRNAs to silence their respective target genes more effectively. However, similar to our previous study, the hexon and protease RNA-targeting siRNAs were relatively poor inhibitors of virus multiplication under these conditions. These results reflect those obtained in experiments in which we inhibited viral DNA synthesis by siRNAs, but were unable to further increase the overall antiviral effect by also targeting the hexon and protease transcripts [22]. Thus, the data presented here support our previous observations that argue against a concomitant targeting of different viral processes (e.g., viral DNA synthesis and capsid generation) as the method of choice to inhibit adenovirus multiplication but, rather, for the targeting of only a single process such as viral DNA synthesis at different steps.

Thus, it was conclusive to incorporate the individual modules for HSV-TK and pTP-mi5 expression into one common vector to concomitantly target adenoviral DNA synthesis at 2 different points. The combinatorial approach revealed an increase in the overall anti-adenoviral effect; however, this effect was modest when we inserted only one copy of the pTP-mi5 sequence into the vector (Figure 4). Because concatemerization of identical amiRNA sequences has been shown to increase knockdown rates [40,41], and concatemerization of pTP-mi5 has also previously resulted in enhanced silencing of Ad5 pTP [21], we added more copies to the vector. We did not observe any detrimental effects on cell viability that could potentially occur due to the higher number of amiRNAs potentially competing with endogenous miRNAs. In case of *in vivo* testing of this and related vectors, this point would have to be carefully addressed, though. The increased additive effects of combined HSV-TK/amiRNA expression obtained with this final vector were most pronounced when GCV was applied at very low concentrations (0.15 μM) (Figure 5). At these low concentrations, inhibition of adenoviral replication mediated by HSV-TK expression alone became marginal (Figure 5A); however, it was restored to normal levels upon concomitant knockdown of pTP gene expression by pTP-mi5. This observed effect is conclusive because, at very low concentrations, GCV-ppp needed for the blockage of DNA polymerization is expected to become limiting when, at the same time, high numbers of viral DNA replication complexes work in parallel to synthesize high numbers of viral DNA molecules. In contrast, the incorporation rate of GCV-ppp into nascent viral DNA strands should increase when only a few viral DNA molecules are generated.

In summary, our data suggest that wt adenovirus DNA replication can, in principle, be blocked most effectively by targeting 2 independent steps needed for viral DNA replication, namely (i) the formation of the initiation complex, and (ii) the actual DNA polymerization step. Targeting of these mechanistically different steps may not be restricted to the way we accomplished it, but may also prove a useful strategy when aiming to inhibit adenoviral DNA replication by novel “conventional” drugs or small-molecule inhibitors. In general, our strategy does not allow to cure wild-type virus-infected cells from the infection, and it cannot prevent them from cell death. However, it can effectively decrease the output of infectious virus progeny from these cells, and consequently prevent virus spreading.

In an envisioned therapeutic scenario, the delivery of anti-adenoviral amiRNAs, via a replication-deficient adenoviral vector, may have several unique advantages. For example, it may allow for the amplification of amiRNA and HSV-TK expression cassette copy numbers upon exposure of the recombinant virus to the wt virus due to the initiation of vector replication. This transcomplementation effect has already been demonstrated earlier for the pTP-mi5 amiRNA expression cassette *in vitro*[21]. In addition, this effect may ensure a constant supply of recombinant vector as long as wt adenovirus is present. Moreover, due to the shared organ tropism of the adenoviral vector and its wt counterpart, delivery via an adenovirus-based vector may also permit the directing of the vector predominantly to those cells and organs that are also the preferred targets of the wt virus. Among those is the liver which functions as a major virion multiplicator during adenovirus infection and is readily transduced by adenoviral vectors [42]. Thus, it is conceivable, that strategies as the one presented here are able to reduce the output of infectious wt virus at least from this particular organ, and, consequently, inhibit spreading of the virus throughout the body. Because the immune response against the wild-type virus is heavily impaired or inexistent in immunocompromised patients, problems that can occur due to the elimination of adenoviral vectors by the immune system should be less pronounced or absent in such an envisioned scenario. Alternatively, topical treatment of localized adenovirus infection, e.g., of the eye, where high local vector concentrations can be achieved, is conceivable for immunocompetent patients as well. Adenovirus-mediated epidemic keratoconjunctivitis (EKC) is associated with significant morbidity and possible long term consequences on visual acuity, and thus causes considerable economic losses.

Adenoviral vectors are among the most commonly used vectors for the delivery of genetic information *in vivo*[42]. In most cases, replication-deficient vectors whose E1 and E3 genes are deleted are employed. In theory, no viral genes should be expressed from these vectors. Leaky expression of viral genes from replication-deficient viral vectors, however, is known to occur due to the presence of binding sites for certain cellular transcription factors in adenoviral promoters [43]. This background expression from both early and late promoters can result in toxicity and immunity against adenoviral proteins [44-48] and, consequently, to short-lived transgene expression. The amiRNA generated from the combinatorial HSV-TK/amiRNA expression cassette, when included in such vectors, may potentially prevent leaky pTP expression from the adenoviral E2B promoter. It may, thus, inhibit any potential low-level replication and, consequently, late gene expression (which is dependent on viral DNA replication), thereby preventing the generation of highly immunogenic late gene products such as the hexon and fiber proteins. In fact, deletions within the E2B region that comprises the viral DNA polymerase and pTP genes lead to decreased downstream gene expression in E1-deleted adenoviral vectors [49,50], resulting in extended transgene expression and reduced liver toxicity [50,51].

The HSV-TK expression unit of the cassette, in conjunction with the amiRNA expression unit, may also help to bring any unforeseen high-level replication of adenoviral vectors under control, should they turn into replication-competent versions (i.e., upon unintended recombination with a wt virus). The elimination upon treatment with GCV would only affect the rearranged vector and not the parental vector because the latter would not promote the synthesis of significant amounts of HSV-TK. Thus, the combinatorial gene expression cassette presented here (or similar versions following the same principle) may also be considered for evaluation as safeguards for certain adenoviral vectors intended to be used in gene therapy studies.

## Conclusions

In summary, the results presented in this study demonstrate that combined expression of HSV-TK and an amiRNA targeting the adenoviral pTP mRNA can effectively inhibit wt adenovirus replication in the presence of the antiherpetic prodrug GCV. The enhanced inhibition rate accomplished with the combinatorial gene expression cassette can most likely be attributed to the targeting of 2 different steps in adenoviral DNA synthesis. This effect mediated by the expression cassette may not only be harnessed to inhibit wt adenovirus replication but also to prevent the replication of certain adenoviral vectors commonly used in gene therapy studies.

## Competing interests

The authors declare that they have no competing interests.

## Authors’ contributions

MI performed the experiments, analyzed the data, and participated in the preparation of the manuscript; TL participated in the preparation of the manuscript; RK designed research, analyzed the data, and wrote the manuscript. All authors read and approved the final manuscript.

## References

[B1] Echavarria M Adenoviruses in immunocompromised hosts Clin Microbiol Rev 2008 21 4 704 715 10.1128/CMR.00052-07 18854488 PMC2570151

[B2] Ison MG Adenovirus infections in transplant recipients Clin Infect Dis 2006 43 3 331 339 10.1086/505498 16804849

[B3] Kojaoghlanian T Flomenberg P Horwitz MS The impact of adenovirus infection on the immunocompromised host Rev Med Virol 2003 13 3 155 171 10.1002/rmv.386 12740831

[B4] Blanke C Clark C Broun ER Tricot G Cunningham I Cornetta K Hedderman A Hromas R Evolving pathogens in allogeneic bone marrow transplantation: increased fatal adenoviral infections Am J Med 1995 99 3 326 328 10.1016/S0002-9343(99)80169-7 7653496

[B5] Hale GA Heslop HE Krance RA Brenner MA Jayawardene D Srivastava DK Patrick CC Adenovirus infection after pediatric bone marrow transplantation Bone Marrow Transplant 1999 23 3 277 282 10.1038/sj.bmt.1701563 10084260

[B6] Howard DS Phillips IG Reece DE Munn RK Henslee-Downey J Pittard M Barker M Pomeroy C Adenovirus infections in hematopoietic stem cell transplant recipients Clin Infect Dis 1999 29 6 1494 1501 10.1086/313514 10585802

[B7] Lion T Baumgartinger R Watzinger F Matthes-Martin S Suda M Preuner S Futterknecht B Lawitschka A Peters C Potschger U Molecular monitoring of adenovirus in peripheral blood after allogeneic bone marrow transplantation permits early diagnosis of disseminated disease Blood 2003 102 3 1114 1120 10.1182/blood-2002-07-2152 12702513

[B8] Munoz FM Piedra PA Demmler GJ Disseminated adenovirus disease in immunocompromised and immunocompetent children Clin Infect Dis 1998 27 5 1194 1200 10.1086/514978 9827268

[B9] Ebner K Rauch M Preuner S Lion T Typing of human adenoviruses in specimens from immunosuppressed patients by PCR-fragment length analysis and real-time quantitative PCR J Clin Microbiol 2006 44 8 2808 2815 10.1128/JCM.00048-06 16891496 PMC1594637

[B10] Ebner K Suda M Watzinger F Lion T Molecular detection and quantitative analysis of the entire spectrum of human adenoviruses by a two-reaction real-time PCR assay J Clin Microbiol 2005 43 7 3049 3053 10.1128/JCM.43.7.3049-3053.2005 16000414 PMC1169147

[B11] Lion T Kosulin K Landlinger C Rauch M Preuner S Jugovic D Potschger U Lawitschka A Peters C Fritsch G Monitoring of adenovirus load in stool by real-time PCR permits early detection of impending invasive infection in patients after allogeneic stem cell transplantation Leukemia 2010 24 4 706 714 10.1038/leu.2010.4 20147979

[B12] Lenaerts L De Clercq E Naesens L Clinical features and treatment of adenovirus infections Rev Med Virol 2008 18 6 357 374 10.1002/rmv.589 18655013

[B13] Lindemans CA Leen AM Boelens JJ How I treat adenovirus in hematopoietic stem cell transplant recipients Blood 2010 116 25 5476 5485 10.1182/blood-2010-04-259291 20837781 PMC3031399

[B14] Ljungman P Ribaud P Eyrich M Matthes-Martin S Einsele H Bleakley M Machaczka M Bierings M Bosi A Gratecos N Cidofovir for adenovirus infections after allogeneic hematopoietic stem cell transplantation: a survey by the infectious diseases working party of the European group for blood and marrow transplantation Bone Marrow Transplant 2003 31 6 481 486 10.1038/sj.bmt.1703798 12665844

[B15] Symeonidis N Jakubowski A Pierre-Louis S Jaffe D Pamer E Sepkowitz K O’Reilly RJ Papanicolaou GA Invasive adenoviral infections in T-cell-depleted allogeneic hematopoietic stem cell transplantation: high mortality in the era of cidofovir Transpl Infect Dis 2007 9 2 108 113 10.1111/j.1399-3062.2006.00184.x 17461995

[B16] Yusuf U Hale GA Carr J Gu Z Benaim E Woodard P Kasow KA Horwitz EM Leung W Srivastava DK Cidofovir for the treatment of adenoviral infection in pediatric hematopoietic stem cell transplant patients Transplantation 2006 81 10 1398 1404 10.1097/01.tp.0000209195.95115.8e 16732176

[B17] Feuchtinger T Matthes-Martin S Richard C Lion T Fuhrer M Hamprecht K Handgretinger R Peters C Schuster FR Beck R Safe adoptive transfer of virus-specific T-cell immunity for the treatment of systemic adenovirus infection after allogeneic stem cell transplantation Br J Haematol 2006 134 1 64 76 10.1111/j.1365-2141.2006.06108.x 16803570

[B18] Leen AM Christin A Myers GD Liu H Cruz CR Hanley PJ Kennedy-Nasser AA Leung KS Gee AP Krance RA Cytotoxic T lymphocyte therapy with donor T cells prevents and treats adenovirus and Epstein-Barr virus infections after haploidentical and matched unrelated stem cell transplantation Blood 2009 114 19 4283 4292 10.1182/blood-2009-07-232454 19700662 PMC2774556

[B19] Chung YS Kim MK Lee WJ Kang C Silencing E1A mRNA by RNA interference inhibits adenovirus replication Arch Virol 2007 152 7 1305 1314 10.1007/s00705-007-0951-z 17597352 PMC7087230

[B20] Eckstein A Grossl T Geisler A Wang X Pinkert S Pozzuto T Schwer C Kurreck J Weger S Vetter R Inhibition of adenovirus infections by siRNA-mediated silencing of early and late adenoviral gene functions Antiviral Res 2010 88 1 86 94 10.1016/j.antiviral.2010.08.002 20708037

[B21] Ibrisimovic M Kneidinger D Lion T Klein R An adenoviral vector-based expression and delivery system for the inhibition of wild-type adenovirus replication by artificial microRNAs Antiviral Res 2012 97 1 10 23 23127366 10.1016/j.antiviral.2012.10.008 PMC3552158

[B22] Kneidinger D Ibrisimovic M Lion T Klein R Inhibition of adenovirus multiplication by short interfering RNAs directly or indirectly targeting the viral DNA replication machinery Antiviral Res 2012 94 3 195 207 10.1016/j.antiviral.2012.03.011 22510340 PMC3370646

[B23] Elbashir SM Harborth J Lendeckel W Yalcin A Weber K Tuschl T Duplexes of 21-nucleotide RNAs mediate RNA interference in cultured mammalian cells Nature 2001 411 6836 494 498 10.1038/35078107 11373684

[B24] Cullen BR Derivation and function of small interfering RNAs and microRNAs Virus Res 2004 102 1 3 9 10.1016/j.virusres.2004.01.009 15068874

[B25] Burnett JC Rossi JJ RNA-based therapeutics: current progress and future prospects Chem Biol 2012 19 1 60 71 10.1016/j.chembiol.2011.12.008 22284355 PMC3269031

[B26] Zeng Y Wagner EJ Cullen BR Both natural and designed micro RNAs can inhibit the expression of cognate mRNAs when expressed in human cells Molecular cell 2002 9 6 1327 1333 10.1016/S1097-2765(02)00541-5 12086629

[B27] Davidson BL McCray PB Jr Current prospects for RNA interference-based therapies Nat Rev Genet 2011 12 5 329 340 10.1038/nrg2968 21499294 PMC7097665

[B28] Huntzinger E Izaurralde E Gene silencing by microRNAs: contributions of translational repression and mRNA decay Nat Rev Genet 2011 12 2 99 110 10.1038/nrg2936 21245828

[B29] Ibrisimovic M Nagl U Kneidinger D Rauch M Lion T Klein R Targeted expression of herpes simplex virus thymidine kinase in adenovirus-infected cells reduces virus titers upon treatment with ganciclovir *in vitro* J Gene Med 2012 14 1 3 19 22190534 10.1002/jgm.1638

[B30] Faulds D Heel RC Ganciclovir. A review of its antiviral activity, pharmacokinetic properties and therapeutic efficacy in cytomegalovirus infections Drugs 1990 39 4 597 638 10.2165/00003495-199039040-00008 2161731

[B31] Ilsley DD Lee SH Miller WH Kuchta RD Acyclic guanosine analogs inhibit DNA polymerases alpha, delta, and epsilon with very different potencies and have unique mechanisms of action Biochemistry 1995 34 8 2504 2510 10.1021/bi00008a014 7873530

[B32] Golankiewicz B Ostrowski T Tricyclic nucleoside analogues as antiherpes agents Antiviral Res 2006 71 2–3 134 140 16780965 10.1016/j.antiviral.2006.05.004

[B33] Elion GB Furman PA Fyfe JA de Miranda P Beauchamp L Schaeffer HJ Selectivity of action of an antiherpetic agent, 9-(2-hydroxyethoxymethyl) guanine Proc Natl Acad Sci U S A 1977 74 12 5716 5720 10.1073/pnas.74.12.5716 202961 PMC431864

[B34] Berk AJ Recent lessons in gene expression, cell cycle control, and cell biology from adenovirus Oncogene 2005 24 52 7673 7685 10.1038/sj.onc.1209040 16299528

[B35] Liu F Green MR Promoter targeting by adenovirus E1a through interaction with different cellular DNA-binding domains Nature 1994 368 6471 520 525 10.1038/368520a0 8139685

[B36] Asano-Mori Y Kanda Y Oshima K Watanabe T Shoda E Motokura T Kurokawa M Chiba S Pharmacokinetics of ganciclovir in haematopoietic stem cell transplantation recipients with or without renal impairment J Antimicrob Chemother 2006 57 5 1004 1007 10.1093/jac/dkl089 16551692

[B37] Caldes A Colom H Armendariz Y Garrido MJ Troconiz IF Gil-Vernet S Lloberas N Pou L Peraire C Grinyo JM Population pharmacokinetics of ganciclovir after intravenous ganciclovir and oral valganciclovir administration in solid organ transplant patients infected with cytomegalovirus Antimicrob Agents Chemother 2009 53 11 4816 4824 10.1128/AAC.00085-09 19738014 PMC2772326

[B38] Frenkel LM Capparelli EV Dankner WM Xu J Smith IL Ballow A Culnane M Read JS Thompson M Mohan KM Oral ganciclovir in children: pharmacokinetics, safety, tolerance, and antiviral effects. The Pediatric AIDS Clinical Trials Group J Infect Dis 2000 182 6 1616 1624 10.1086/317600 11069232

[B39] Cundy KC Clinical pharmacokinetics of the antiviral nucleotide analogues cidofovir and adefovir Clin Pharmacokinet 1999 36 2 127 143 10.2165/00003088-199936020-00004 10092959

[B40] Chung KH Hart CC Al-Bassam S Avery A Taylor J Patel PD Vojtek AB Turner DL Polycistronic RNA polymerase II expression vectors for RNA interference based on BIC/miR-155 Nucleic Acids Res 2006 34 7 e53 10.1093/nar/gkl143 16614444 PMC1435982

[B41] Wu Z Xue Y Wang B Du J Jin Q Broad-spectrum antiviral activity of RNA interference against four genotypes of Japanese encephalitis virus based on single microRNA polycistrons PLoS One 2011 6 10 e26304 10.1371/journal.pone.0026304 22028851 PMC3196537

[B42] Khare R Chen CY Weaver EA Barry MA Advances and future challenges in adenoviral vector pharmacology and targeting Current gene therapy 2011 11 4 241 258 10.2174/156652311796150363 21453281 PMC3267160

[B43] Jones NC Rigby PW Ziff EB Trans-acting protein factors and the regulation of eukaryotic transcription: lessons from studies on DNA tumor viruses Genes Dev 1988 2 3 267 281 10.1101/gad.2.3.267 3288540

[B44] Yang Y Nunes FA Berencsi K Gonczol E Engelhardt JF Wilson JM Inactivation of E2a in recombinant adenoviruses improves the prospect for gene therapy in cystic fibrosis Nat Genet 1994 7 3 362 369 10.1038/ng0794-362 7522742

[B45] Yang Y Nunes FA Berencsi K Furth EE Gonczol E Wilson JM Cellular immunity to viral antigens limits E1-deleted adenoviruses for gene therapy Proc Natl Acad Sci U S A 1994 91 10 4407 4411 10.1073/pnas.91.10.4407 8183921 PMC43794

[B46] Engelhardt JF Litzky L Wilson JM Prolonged transgene expression in cotton rat lung with recombinant adenoviruses defective in E2a Hum Gene Ther 1994 5 10 1217 1229 10.1089/hum.1994.5.10-1217 7849095

[B47] Lieber A He CY Meuse L Himeda C Wilson C Kay MA Inhibition of NF-kappaB activation in combination with bcl-2 expression allows for persistence of first-generation adenovirus vectors in the mouse liver J Virol 1998 72 11 9267 9277 9765474 10.1128/jvi.72.11.9267-9277.1998 PMC110346

[B48] Lieber A He CY Meuse L Schowalter D Kirillova I Winther B Kay MA The role of Kupffer cell activation and viral gene expression in early liver toxicity after infusion of recombinant adenovirus vectors J Virol 1997 71 11 8798 8807 9343240 10.1128/jvi.71.11.8798-8807.1997 PMC192346

[B49] Amalfitano A Hauser MA Hu H Serra D Begy CR Chamberlain JS Production and characterization of improved adenovirus vectors with the E1, E2b, and E3 genes deleted J Virol 1998 72 2 926 933 9444984 10.1128/jvi.72.2.926-933.1998 PMC124562

[B50] Everett RS Hodges BL Ding EY Xu F Serra D Amalfitano A Liver toxicities typically induced by first-generation adenoviral vectors can be reduced by use of E1, E2b-deleted adenoviral vectors Hum Gene Ther 2003 14 18 1715 1726 10.1089/104303403322611737 14670123

[B51] Hodges BL Serra D Hu H Begy CA Chamberlain JS Amalfitano A Multiply deleted [E1, polymerase-, and pTP-] adenovirus vector persists despite deletion of the preterminal protein J Gene Med 2000 2 4 250 259 10.1002/1521-2254(200007/08)2:4<250::AID-JGM113>3.0.CO;2-3 10953916

